# Clinical Features and Risk Stratification of Multiple Myeloma Patients with COVID-19

**DOI:** 10.3390/cancers15143598

**Published:** 2023-07-13

**Authors:** Ruifang Zheng, Kelsey Mieth, Christen Bennett, Carol Miller, Larry D. Anderson, Mingyi Chen, Jing Cao

**Affiliations:** 1 Department of Pathology, University of Texas Southwestern Medical Center, Dallas, TX 75390, USA; 2 Department of Internal Medicine, Division of Hematology and Oncology, University of Texas Southwestern Medical Center, Dallas, TX 75390, USA

**Keywords:** multiple myeloma, COVID-19, SARS-CoV-2, neutralizing antibody, anti-CD38 antibody

## Abstract

**Simple Summary:**

This study is about how COVID-19 affects patients with multiple myeloma (MM), a type of cancer, and aims to provide guidance for managing their risk and treatment. Authors reviewed published data and analyzed the medical records of 34 MM patients who tested positive for the SARS-CoV-2 virus in order to study the impact of chemotherapy and antibody drugs on hospitalization and death rates, as well as patients’ ability to produce antibodies. The findings revealed that MM patients have a higher risk of hospitalization and death from COVID-19, particularly those undergoing active chemotherapy. Older age, high-risk myeloma, renal disease, and poor disease control are predictors of worse outcomes. The use of daratumumab did not worsen the disease or increase hospitalization rates, and neutralizing antibodies were associated with reduced mortality. The study suggests that testing for neutralizing antibodies after COVID-19 vaccination in MM patients is needed to manage their risk of contracting COVID-19.

**Abstract:**

SARS-CoV-2 infection often results in a more severe COVID-19 disease course in multiple myeloma (MM) patients compared to immunocompetent individuals. The aim of this report is to summarize the clinical features of the MM patients with COVID-19 and the impact of MM treatment on outcomes to guide risk stratification and ensure the appropriate management of the patients. Serological responses in MM patients post-infection or -vaccination are also reviewed to better understand the strategy of prevention. Along with reports from the literature, we presented findings from a retrospective analysis of the clinical characteristics and outcomes of COVID-19 infection in MM patients in our institution. Study population includes 34 MM patients with a median age of 61 (range: 35–82 years) who tested positive for SARS-CoV-2 between 1 March 2020–15 August 2021. We examined the effect of chemotherapy, the benefit of neutralizing monoclonal antibody (Bamlanivimab) and the impact of anti-CD38 antibody (daratumumab) on the hospitalization and mortality of the patients, as well as the efficacy of native antibody production. Our results showed that MM patients have increased hospitalization and mortality rates from COVID-19 compared with that of general population, especially those on active chemotherapy. Advanced age, high-risk myeloma, renal disease, and suboptimal disease control are independent predictors of adverse outcomes. The use of daratumumab does not increase the disease severity/hospitalization or the post-infection/vaccination seropositivity of SARS-CoV-2. The neutralizing antibody decreases overall mortality. Evidence from the current study and previous publications suggest that testing of neutralizing antibody post-SARS-CoV-2 vaccination in MM patients may be needed in reducing COVID-19 risk.

## 1. Introduction

Since its initial outbreak in the winter the 2019, there have been multiple rounds of the COVID-19 pandemic, with an estimation of 90% of the world’s population infected by SARS-CoV-2 at least once [1]. The spectrum of symptoms and the severity of the disease vary in the general population. Conversely, in patients with multiple myeloma (MM), an overwhelming majority experience significant systematic deterioration. Recognizing the clinical features and prognostic risk factors of SARS-CoV-2 infection in MM is essential in the management of these patients, given that the virus likely will continue to circulate around the word as a perennial rather than transient threat.

Infection remains the primary cause of morbidity and mortality in MM patients. Therefore, from early phase of the COVID pandemic, there has been strong interest in the presentation of COVID-19 in MM patients. Patients with MM are in dual jeopardy to SARS-CoV-2 infections as a result of intrinsic and therapy-related immunosuppression, older age, and the presence of comorbidities. With a better understanding of the disease course and characteristics, further interest has emerged in the impact of MM therapy on COVID progression, and the serological response to SARS-CoV-2 infection or vaccination in MGUS and myeloma patients. More recently, specific recommendations regarding risk assessment and treatment prioritization were published by expert groups in a number of countries.

This report elaborates on the following four aspects to summarize evidence on managing MM patients with COVID-19, namely, the clinical features of MGUS and myeloma patients with SARS-CoV-2 infection, the impact of MM treatment on COVID prognosis, the serological responses in MGUS and myeloma patients post-infection or vaccination, and ultimately, recommendations on managing MM patients with COVID. Data from a retrospective study in our institution are presented too. We examined the clinical features and the effect of treatment in MM patients diagnosed with COVID-19.

## 2. Clinical Features of MM Patients with SARS-CoV-2 Infection

In a retrospective study conducted by Wang et al. [2], a total of 58 patients, including 54 with MM and 4 with smoldering multiple myeloma (SMM), that had contracted COVID-19 between March and April 2020 were analyzed. The study revealed that hypertension, hyperlipidemia, and obesity were the most prevalent comorbidities, affecting over 30% of the cohort. Among the 58 patients, 24% succumbed to the infection. The factors significantly associated with hospitalization included age over 70 years, male gender, cardiovascular disease, and not being in complete or stringent complete remission. Inflammatory markers such as C-reactive protein (CRP), ferritin, and D-dimer were found to be elevated in hospitalized patients and were associated with increased mortality. Additionally, severe hypogammaglobulinemia and non-white race were significantly associated with higher mortality.

The International Myeloma Society conducted a retrospective study involving 650 hospitalized patients with plasma cell disorders who were diagnosed with COVID-19 [3]. The results showed that COVID-19 led to moderate-to-severe respiratory dysfunction in 75% to 80% of MM patients, with a death rate ranging from 27% to 57% among hospitalized MM patients with COVID-19. A multivariate analysis identified factors such as age, high-risk MM, renal disease, and suboptimal MM control as being associated with unfavorable mortality outcomes. However, no significant link was found between transplant history or MM medication usage and mortality.

Another research effort aimed to evaluate the global impact of COVID-19 on MM patients by utilizing a federated data network called TriNetX, which provided access to electronic medical records from healthcare organizations worldwide [4]. Through propensity score-matched analysis, the study observed a decrease in the number of new MM diagnoses in 2020 compared to 2019 (relative risk: RR 0.86; 95% confidence interval: CI 0.76–0.96) and a reduction in the survival rate of newly diagnosed MM cases (hazard ratio: HR 0.61; 95% CI: 0.38–0.81). Additionally, MM patients were found to have an increased risk of contracting SARS-CoV-2 (RR: 2.09; 95% CI: 1.58–2.76) and a higher excess mortality rate in 2020 compared to non-MM patients (difference in excess mortality: 9%; 95%: CI 4.4–13.2%).

In our own retrospective study conducted at a single center, we collected data from MM patients who tested positive for SARS-CoV-2. The study protocol was approved by the Institutional Review Board as exempt due to its retrospective nature. We compared the clinical features and effect of treatment in MM patients using the Chi-square probability test. A total of 34 MM patients were included in the study, all of whom tested positive for SARS-CoV-2 between 1 March 2020 and 15 August 2021 (Table 1). The median age of the patients was 61 years, ranging from 35 to 82 years. Among them, 10 patients (29%) required hospitalization due to the infection, and tragically, 2 patients (6%) succumbed to the illness. By utilizing demographic, clinical, and laboratory parameters commonly available in MM patients, another study implemented machine learning algorithms [5] to create a multivariable predictive model for COVID-19 outcomes in MM patients. This model holds the potential to aid in risk stratification and provide earlier guidance for clinical decision making.

In contrast to the markedly increased risk from SARS-CoV-2 infection in myeloma patients, a recent retrospective study [6] examined the prevalence of COVID-19 among a group of 1454 patients with MGUS. Out of the 91 patients who tested positive for the virus using RT-PCR on nasopharyngeal swabs between 1 March 2020 and 30 April 2021, the authors found that those with MGUS were equally susceptible to the infection and had similar outcomes as healthy controls of the same age and gender during the initial wave of the COVID-19 pandemic.

## 3. Impact of MM Treatment on COVID Prognosis

In addition to the impact of the serological response to vaccination, MM treatment itself has been found to influence the course and outcomes of COVID-19 in patients. Various studies have shed light on the association between specific MM therapies and the risk of COVID-19 infection, as well as the severity of the disease.

The management of MM in the post COVID-19 era requires a careful balance between effective MM treatment and minimizing infection to optimize outcomes. The immunosuppressive effects of MM therapy may contribute to the higher risk of severe COVID-19 in these patients. In Wang et al. [2], MM disease status and treatment at the time of contracting COVID-19 had no impact on mortality. A meta-analysis conducted by Vijenthira [7] examined the outcomes of MM patients who were diagnosed with COVID-19. The study found that patients who were receiving active MM therapy had a higher risk of developing severe COVID-19 symptoms, including acute respiratory distress syndrome (ARDS), and requiring mechanical ventilation. However, patients who were in remission or not receiving active treatment had a similar risk of severe COVID-19 as the general population. A retrospective study [8] with a total of 740 patients found that inflammatory mediators such as C-reactive protein (CRP), ferritin, and lactate dehydrogenase (LDH) showed a statistically significant difference between the Tocilizumab group and the control group. There is no statistically significant difference in the primary outcome (discharged alive or death) or secondary outcome (length of hospital stay) between patients treated with Tocilizumab and the control group.

In our single-center study, we analyzed the effect of chemotherapy, the potential benefits of neutralizing monoclonal antibody (Bamlanivimab) and anti-CD38 antibody (daratumumab) to the hospitalization and mortality of MM patients, as well as the efficacy of native protective antibody production (Table 1). Patients on active chemotherapy (N = 10) had a higher rate of hospitalization and mortality compared to those on low-dose maintenance or free of therapy, 60% vs. 16.7%, and 20% vs. 0%, respectively. The use of daratumumab showed no significant effect on hospitalization and mortality, with rates of 36.4% vs. 26.1%, and 9.0% vs. 4.3%, respectively. The SARS-CoV-2 neutralizing antibody (Bamlanivimab) was given to the high-risk patients in our institution, and the rate of hospitalization was not affected, with a rate of 30% (with antibody) vs. 29% (without antibody). However, the neutralizing antibody decreased the mortality rate, with 8% vs. 0%.

One important aspect to consider is the use of immunomodulatory drugs (IMiDs) such as lenalidomide and pomalidomide in MM treatment. IMiDs have been shown to possess immunomodulatory and anti-inflammatory properties that may have implications for the immune response to SARS-CoV-2. Chari et al. investigated the impact of lenalidomide maintenance therapy on COVID-19 outcomes in MM patients [3]. The results demonstrated that lenalidomide treatment was not associated with an increased risk of COVID-19 infection or severe disease. These findings suggest that certain MM therapies, like lenalidomide, may not negatively impact the immune response to SARS-CoV-2.

On the other hand, the use of proteasome inhibitors, such as bortezomib and carfilzomib, in MM treatment has raised concerns regarding their potential immunosuppressive effects. Proteasome inhibitors have been reported to impair the function of T cells and natural killer cells, which are crucial components of the immune response against viral infections. In retrospective studies evaluating the impact of bortezomib-based therapy on COVID-19 outcomes in MM patients, the results indicated that patients who received bortezomib close to COVID-19 diagnosis (within a month) had a significantly higher risk of severe disease compared to those who did not receive bortezomib [9,10]. These findings suggest that caution should have been exercised when considering the use of proteasome inhibitors in MM patients during the COVID-19 pandemic.

Furthermore, the use of monoclonal antibodies, such as daratumumab, to target CD38 has become an integral part of MM treatment. However, these antibodies can deplete normal plasma cells, which may compromise the humoral immune response to SARS-CoV-2. A study examined the impact of daratumumab treatment on COVID-19 outcomes in MM patients [2]. The findings revealed that patients who had received daratumumab within three months prior to COVID-19 diagnosis had a higher risk of hospitalization compared to those who did not receive daratumumab. These results emphasize the need for careful consideration of the timing and duration of daratumumab therapy in MM patients during the ongoing pandemic.

The impact of MM treatment on COVID-19 outcomes is a complex and multifaceted issue. While some therapies, such as lenalidomide, may not negatively affect the immune response to SARS-CoV-2, others, like treatment with proteasome inhibitors and CD38-targeting monoclonal antibodies, have been associated with an increased risk of severe disease. It is crucial for healthcare providers to carefully evaluate the risks and benefits of specific MM treatments in the context of the COVID-19 pandemic, taking into consideration the individual patient’s disease status, treatment response, and overall immune function. Close monitoring and proactive management of COVID-19 in MM patients can help to optimize outcomes and reduce the burden of this infectious disease in this vulnerable population.

## 4. Serological Responses in MGUS and Myeloma Patients Post Infection or Vaccination

Prior to the COVID-19 pandemic, studies conducted on patients with monoclonal gammopathy of undetermined significance (MGUS) and healthy controls did not find significant differences in the antiviral immunological response to herpes viruses, cytomegalovirus, or Epstein–Barr virus. However, it was observed that the median titer of anti-varicella-zoster virus IgG was significantly lower in MGUS patients [11]. This led to speculation that the intrinsic immune dysregulation in MGUS patients might contribute to a suboptimal serological response to vaccines, including those for SARS-CoV-2.

In the context of SARS-CoV-2 vaccination, studies have shown that the response in neutralizing antibodies in fully vaccinated patients with precursor stages of multiple myeloma (MM) was comparable to that of healthy controls and asymptomatic patients [12]. Another study investigated the efficacy of anti-SARS-CoV-2 vaccines in MGUS patients and found that fully vaccinated individuals had a similar likelihood of SARS-CoV-2 infection compared to unvaccinated MGUS patients, but experienced improved COVID-19 outcomes [13].

In contrast to MGUS patients, MM patients face an extremely high risk when it comes to COVID-19 due to intrinsic and therapy-related immunosuppression, older age, and the presence of comorbidities. Van Oekelen et al. [14] conducted a study involving 260 fully immunized MM patients and found that 16% of them did not have detectable IgG antibody titers against the SARS-CoV-2 spike receptor binding domain following the second mRNA vaccine dose, even after a median of 51 days. In comparison, all control patients with similar age and gender distribution had detectable anti-SARS-CoV-2 antibodies. This reduced antibody response in MM patients is expected since MM treatments specifically target the plasma cells responsible for antibody production. Among the 41 patients who showed no antibody response, 58.5% were undergoing anti-CD38 antibody therapy during vaccination, 31.7% were receiving anti-BCMA bispecific antibody treatment, and 9.8% had undergone anti-BCMA CAR-T therapy in the three months prior [14].

Similar findings were reported by Terpos et al., who evaluated the presence of neutralizing antibodies in 213 symptomatic MM patients vaccinated with either the BNT162b2 or AZD1222 vaccines. A significant proportion of MM patients, especially those undergoing treatment with anti-CD38 or anti-BCMA therapies, did not develop anti-SARS-CoV-2 antibodies or had insufficient serological responses even after receiving two vaccine doses [15]. A neutralizing antibody titer of 50% or higher is considered clinically effective and is the target titer in the majority of vaccine trials [16]. However, on day 50 after the first dose, only 53.5% of MM patients had a neutralizing antibody titer higher than 50%, compared to 81% in controls [15].

The aforementioned studies raised concerns that, although there was no evidence supporting this concern in MGUS patients, patients with SMM and MM may have a weakened antibody response compared to healthy controls, thus validating the need for at least two doses and a booster anti-SARS-CoV-2 vaccine [15,17,18,19]. In our study cohort, 15 patients underwent antibody screening after infection or two doses of vaccine (BNT162b2 or mRNA-1273), 73% (11/15) of them showed seropositivity, lower than the reported 97.5% in the healthy population. There was no significant difference in seropositivity between patients treated with or without daratumumab, 75% vs. 73% (Table 1). A major limitation of the study from our institution is the limited number of patients, particularly the small number undergoing active treatment. Nevertheless, our data align with evidence from the literature that MM patients had a lower rate of anti-SARS-CoV-2 seropositivity than healthy counterparts.

## 5. Recommendations on Managing MM Patients with COVID

As COVID-19 continues to evolve into a persistent public health concern, the importance of effective SARS-CoV-2 vaccination in patients with multiple myeloma (MM) cannot be overstated. Several studies emphasize the need to monitor antibody titers following vaccination in MM patients [19,20]. These patients face a dual challenge as those most likely to have a poor vaccination response, such as those on anti-CD38 or anti-BCMA-based therapies, are also at the highest risk of developing severe COVID-19. Monitoring neutralizing antibody titers can help to identify these patients and personalize risk reduction plans, especially as mask and social distancing restrictions are eliminated globally.

While spike receptor-binding domain IgG antibodies have been correlated with neutralizing antibody titers, the actual measurement of neutralizing antibodies provides a better reflection of humoral immunity against the virus. Recent data indicate that neutralization levels against SARS-CoV-2 are highly predictive of immune protection. Testing for neutralizing antibodies is recommended around four weeks after completing COVID-19 vaccination. A neutralization level of 20.2% (95% CI, 14.4–28.4%) of the mean convalescent level has been estimated to provide 50% protection against detectable SARS-CoV-2 infection [21]. Additionally, data on T-cell immunity after vaccination in MM patients and its potential protection against COVID-19 are accumulating [19]. However, until sufficient evidence is available, MM patients with suboptimal neutralizing antibody responses are advised to continue practicing precautions such as wearing masks and maintaining social distancing.

Regarding booster vaccine doses in immunocompromised individuals, including MM patients, limited data are available. The CDC Advisory Committee on Immunization Practices has recommended booster COVID-19 vaccines for patients with chronic medical conditions, HIV infection, immunocompromised states, and cancer. However, specific data on immune responses to booster vaccinations, particularly in patients with hematologic malignancies undergoing active chemotherapy, as well as anti-CD38 or anti-BCMA regimens, are lacking. Although post-vaccination COVID-19 titer testing is not required for the general population, it is considered essential for risk stratification in special patient populations with severe immunosuppression, such as those with chronic lymphocytic leukemia, lymphoma, or MM on anti-CD20-based regimens or Bruton’s tyrosine kinase inhibitors [22].

To address the management of MM patients with COVID-19, a panel of experts from the Brazilian Association of Hematology, Hemotherapy and Cell Therapy (ABHH) Monoclonal Gammopathies Committee published guidelines on the challenges of MM diagnosis and therapy during the pandemic. For autologous stem-cell transplant (ASCT) candidates who have experienced COVID-19 symptoms with laboratory confirmation, the transplant should be delayed for three months, or a minimum of 21 days if ASCT is considered a priority [4]. The European Society for Medical Oncology (ESMO) has also provided recommendations for prioritizing COVID-19 patients with monoclonal gammopathy of undetermined significance (MGUS) or myeloma [23]. Additionally, recommendations from clinicians in the US are available [24].

Monitoring antibody titers and neutralizing antibodies following vaccination is crucial in MM patients to assess immune response and personalize risk reduction plans. While data on booster vaccinations in this patient population are limited, testing for neutralizing antibodies may help in risk stratification. Recommendations for managing MM patients during the COVID-19 pandemic, including guidelines for autologous stem-cell transplant candidates and prioritization strategies, have been proposed by various expert committees. As our understanding of COVID-19 and its impact on MM patients continues to evolve, ongoing research and multidisciplinary collaboration will be essential to optimizing patient care and outcomes.

## 6. Conclusions

Clinical manifestations of COVID-19 are variable, ranging from a complete absence of symptoms to severe pneumonia, multi-organ failure, and death. The primary risk factors for a poor outcome of COVID-19 include advanced age and comorbidities, conditions that often occur in patients with MM.

The literature reviewed in this article and results from our institution demonstrate that MM patients have increased hospitalization and mortality due to SARS-CoV-2 infection, compared with the rate of the general population. This is especially the case for those receiving active chemotherapy. Advanced age, high-risk myeloma, renal disease, and suboptimal disease control remain independent predictors of adverse outcomes with COVID-19. The use of daratumumab does not increase the disease severity/hospitalization and the post-infection/vaccination seropositivity of SARS-CoV-2. The neutralizing antibody appears to have little effect in terms of decreasing disease severity/hospitalization, but it possibly decreases overall mortality. The testing of neutralizing antibody post-SARS-CoV-2 vaccination in MM patients is crucial in the preventative management and balancing between reducing COVID-19 risk and the need for MM treatment.

By examining these aspects, we hope to shed light on the management strategies that can be employed for MM patients to prepare for future COVID-19 encounters. The findings will help healthcare professionals to make informed decisions regarding risk assessment, treatment choices, and monitoring protocols. Furthermore, the data from our institution’s study contribute to the growing body of evidence and aid in the development of comprehensive guidelines for the management of MM patients in the context of COVID-19.

In conclusion, MM patients face unique challenges when it comes to SARS-CoV-2 infection due to their underlying disease-, age-, and treatment-related factors. Understanding the clinical manifestations, impact of therapy, and serological responses in these patients is crucial for optimizing their care. The evidence presented in this report, including data from our institution, contributes to the growing knowledge base and provides guidance for managing MM patients during the ongoing COVID-19 pandemic. With continued research and collaboration, we can improve outcomes and reduce the burden of COVID-19 in this vulnerable population.

## Figures and Tables

**Table 1:** Patient demographic information, diagnosis of M component disease, therapy, treatment phase, and co-morbidity. Abbreviations: VRD: velcade/lenalidomide/dexamethasone; KRD: kyprolis/lenalidomide/dexamethasone; Rev: lenalidomide; Dex: dexamethasone; Dara: daratumumab Rev/Dex: revlimid/dexamethasone; MM: multiple myeloma; POEMS: POEMS syndrome (osteosclerotic myeloma); MRD: minimal residual disease.

Patient ID	Age	Covid Ab	Diagnosis	MM Stage (R-ISS)	Disease State When Tested COVID-19 Positive	Prior Chemo Therapy	Active Chemo Therapy When Tested COVID-19 Positive (Yes/No/Maintenance)	Stem Cell Transplant before COVID-19 Infection (Yes/No)	Co-Morbidities	Hospitalization Due to COVID-19 Infection	Death Due to COVID-19 Infection	Bamlanivimab (Yes/No)
1	58	Negative	MM	Stage I	Persistent disease (3.9% plasma cells)	Cytoxan/Lenalidomide/Dex, Dara	No	No	T2DM, HLD	No	No	No
2	58	NA	MM	Stage I	In remission	VRD	No	Yes	CHF, HTN, T2DM	No	No	No
3	63	NA	MM	Unknown	In remission	Bortezomib/Rev/Dex	No	Yes	Rheumatiod Arthritis	no	No	No
4	74	NA	MM	Unknown	Persistent disease (5–10% plasma cells)	Dara/Pomalidomide/Dex	No	No	Bladder cancer: s/p local therapy; prostate cancer: s/p radiation therapy	No	No	No
5	69	Negative	MM	Stage I	MRD (<5% plasma cells)	VRD	Yes (pomalidomide)	No	HTN, basal cell carcinoma, GERD, follicular lymphoma (grade II)	Yes (intubation)	No	No
6	46	Postive	MM	Stage I	In remsssion	KRD	Maintenance (Lenalidomide)	Yes	None	No	No	Yes
7	71	NA	MM	Unknown	Newly diagnosed (30% plasma cells)	Not started yet	No	No	HTN	No	No	No
8	66	NA	MM	Stage II	In remission	VRD	No	Yes	None	No	No	Yes
9	52	NA	MM	Unknown	In remission	VRD	Maintenance (Lenalidomide)	Yes	Breast cancer S/P mastectomy and adjuvant chemo; thyroid carcinoma s/p total thyroidectomy	No	No	Yes
10	60	Negative	MM	Stage I	Residual disease (5% plasma cells)	VRD	Maintenance (Lenalidomide)	Yes	None	No	No	No
11	55	NA	MM	Unknown	Residual disease (4% plasma cells)	VRD, Dara	Yes (Dara/Kyprolis/Dex)	Yes	None	No	No	No
12	63	NA	MM	Unknown	In remission	VRD, Dara	Maintenance (Lenalidomide)	Yes	None	No	No	No
13	60	NA	MM	Unknown	In remission	Melphalan	Maintenance (Lenalidomide)	Yes	HTN, T2DM, end stage renal disease	No	No	Yes
14	74	NA	MM	Stage II	Residual disease (1% plasma cells)	VRD	Yes (Dara/Len/Dex)	No	CVA, HTN, HLD, T2DM	No	No	Yes
15	71	Postive	MM	Unknown	In remission	KRD	Yes (KRD)	No	DCIS s/p lympectomy	No	No	Yes
16	54	NA	MM	Stage I	In remission	VRD, Dara	Maintenance (Lenalidomide)	Yes	HTN	No	No	No
17	49	Positive	MM	Stage III	Residual disease (2% plasma cells)	VRD	No	No	HTN	No	No	No
18	35	NA	POEMS	Unknown	In remission	Rev/Dex	No	Yes	Chronic inflammatory demyelinating polyneuropathy	No	No	Yes
19	63	Positive	MM	Unknown	In remission	Velcade/Dex/Dara	No	Yes	HTN	No	No	No
20	50	Positive	MM	Stage II	In remission	VRD, Dara	Maintenance (Lenalidomide)	Yes	Athritis	No	No	No
21	61	NA	MM	Stage II	Near complete remission	KRD	Maintenance (Lenalidomide)	Yes	None	No	No	No
22	71	Positive	MM	Stage I	In remission	VRD	Yes (Rev/Dex)	No	Osteoarthritis	No	No	No
23	59	NA	Amyloidosis	Unknown	Progressive (on onpattro)	None	None	No	End stage infiltrative cardiomyopathy	No	No	No
24	81	Positive	MM	Stage III	Residual disease (unknown plasma cell %)	Ninlaro/Rev/Dex, Dara	Yes (Ninlaro/Rev/Dex)	No	None	Yes	No	No
25	55	NA	MM	Stage II	MRD (0.5% plasma cells)	VRD	Maintenance (Lenalidomide)	Yes	None	No	No	No
26	64	Positive	POEMS	Stage II	In remission	Rev/Dex	Maintenance (Lenalidomide)	Yes	None	Yes	No	No
27	56	NA	MM	Stage III	Near complete remission	VRD	None	No	None	No	No	No
28	82	Negative	MM	Unknown	In remission	Thalidomide/ Dex, Daratumumab	Blenrep	Yes	CM/HF s/p pacemaker, CAD s/p stents, stage 3 CKD	Yes	No	Yes
29	58	Positive	MM	Stage III	In remission	KRD	Maintenance (Lenalidomide)	Yes	RCC	Yes	No	Yes
30	67	Positive	MM	Stage II	Persistent disease (6% plasma cells)	Dara-VRD	Yes (Dara-VRD)	NO	None	Yes	No	Yes
31	55	NA	POEMS	Unknown	Low level disease	Rev/Dex	Yes (Rev/Dex)	No	None	Yes	No	No
32	35	NA	MM (IgD type)	Stage I	MRD (0.3% plasma cells)	VRD	Maintenance (Lenalidomide)	Yes	None	Yes	No	No
33	80	Positive	MM	Stage II	Persistent disease (unknown plasma cell percentage)	Ninlaro/Rev/Dex, Dara	Yes (Dara/Vel/dex)	No	Multi-organ system AL amyloidosis	Yes	Yes	No
34	82	NA	MM	Stage III	Persistent disease (unknown plasma cell percentage)	Cytoxan/Kyprolis/Dex	Yes (Cytoxan/Kyprolis/Dex)	Yes	CHF	Yes	Yes	No

## Data Availability

The data presented in this study are available on request from the corresponding author. The data are not publicly available due to restrictions on study participants’ privacy.
